# The effect of non-invasive transcranial focused ultrasound for depression on the default mode network: an open-label pilot trial

**DOI:** 10.3389/fpsyt.2025.1722575

**Published:** 2026-01-20

**Authors:** Jessica N. Schachtner, Jacob F. Dahill-Fuchel, Diheng Zhang, Katja E. Allen, Christopher R. Bawiec, Peter J. Hollender, Sarah B. Ornellas, Soren D. Konecky, Achal S. Achrol, John J. B. Allen

**Affiliations:** 1 Psychology Department, Psychophysiology Lab, University of Arizona, Tucson, AZ, United States; 2 Openwater, San Francisco, CA, United States

**Keywords:** transcranial-focused ultrasound, default mode network, major depressive disorder, repetitive negative thinking (RNT), neuromodulation

## Abstract

**Background:**

Major depressive disorder (MDD) affects one in five individuals, often recurs, and up to 50% of cases are deemed treatment resistant. Aberrant brain connectivity is associated with both depression symptoms and a thought pattern characteristic of depression, repetitive negative thought (RNT). Transcranial focused ultrasound (tFUS) is a novel neuromodulation technique that can directly target a hypothesized neural mechanism in depression, default mode network (DMN) hyperconnectivity. The present study assessed whether tFUS decreases DMN connectivity in individuals with MDD. Exploratory analyses assessed whether changes in DMN connectivity tracked changes in depressive symptoms and RNT.

**Methods:**

Twenty participants with MDD completed up to 11 sessions of tFUS treatment targeting the left anterior medial prefrontal cortex, a major hub of the DMN. Before commencing and after completing treatment, participants completed resting state functional magnetic resonance imaging, self -report assessments, and clinical interviews. Participants also completed daily self-report and adverse event assessments.

**Results:**

We previously reported a significant decrease in depression symptoms and RNT after tFUS treatment. Here we report that DMN connectivity between the left medial prefrontal cortex and left posterior cingulate cortex, major hubs of the DMN, significantly decreased after treatment. Exploratory analysis revealed no significant relationship between change in DMN connectivity and change in depressive symptoms or RNT.

**Conclusions:**

tFUS shows promise in the treatment for MDD, as hyperconnectivity within the DMN decreased, alongside decreases in depression symptoms and RNT. These findings provide evidence supporting future clinical trials.

**Clinical trial registration:**

https://clinicaltrials.gov/study/NCT06320028, identifier NCT06320028.

## Introduction

The default mode network (DMN) comprises distinct brain regions that are co-active during self-referential processing (e.g., internal mentation, future planning) (1, 2). Previous work consistently identifies the prefrontal cortex (PFC) and posterior cingulate cortex (PCC) as key contributors to DMN self-referential processes due to their robust central connectivity with, and influence on, other hubs of the DMN (1, 3, 4), as well as their strong involvement in integrating information between the DMN and other higher-order cognitive and emotional networks (5). Research suggests that these two regions make up the major hub of the DMN (i.e., subsystem A) and play a critical role in autobiographical decision-making and regulation of other subsystem processes such as episodic judgement and imagery (1).

Many psychiatric disorders are characterized by aberrant DMN function, including major depressive disorder (MDD) (5). The DMN is hyperconnected in depressed individuals (6, 7) and is also associated with *negative* self-referential processing, a specific instance of repetitive negative thought (RNT) (8), which contributes to the maintenance of depression (9). Given the relationship between the DMN, RNT, and MDD, and the fact that up to 50% of individuals with depression are resistant to current treatment options (10), there is a dire need for the development of novel interventions that target specific hypothesized mechanisms of depression, such as hyperconnectivity within the DMN.

Transcranial Focused Ultrasound (tFUS), a non-invasive neuromodulation technique, holds promise as an alternative treatment for MDD. With its ability to target deep brain structures with temporal and spatial precision (11), tFUS may be a better treatment option compared to other neuromodulation techniques (e.g., transcranial magnetic stimulation (TMS), Transcranial Direct Current Stimulation (TDCS)) due to its ability to pass safely through the skull without notable side effects seen from other methods (e.g., itching, heat/burning, and skin irritation (12, 13)). Exploring whether tFUS can reduce depression symptoms and RNT by directly modulating a potential neural mechanism (i.e., the DMN) is a critical next step for improving treatments for MDD.

Few studies have explored modulating brain connectivity to improve clinical symptoms using tFUS. Studies with healthy participants exploring the effects of targeting the inferior frontal gyrus (IFG) of the central executive network (CEN) and the PCC of the DMN found significant reductions in DMN connectivity and improvements in mood (14, 15). In clinically depressed individuals, tFUS targeting the IFG and dorsolateral PFC (DLPFC) of the CEN resulted in mood improvements and DMN connectivity changes (16). Resnik and colleagues found that tFUS targeting the IFG significantly reduced worry in clinically depressed individuals (17).

Given that the PFC is a key hub of the DMN that relates to negative self-referential thought and influences DMN function and integration with other networks (i.e., CEN) (1, 5), as well as its aberrant function in MDD, this region appears to be a promising tFUS target for altering DMN connectivity to treat depression. The present study observed the influence of tFUS targeting the left amPFC on DMN connectivity in individuals with MDD, assessing potential DMN changes following completion of treatment. Although symptomatic improvement in this sample was reported elsewhere (13), this manuscript presents exploratory analysis of resting-state functional magnetic resonance imaging (fMRI) aimed to assess the relationship between pre-to-post changes in DMN connectivity with pre-to-post changes in depression symptoms and RNT.

## Materials and methods

**Figure 1:**
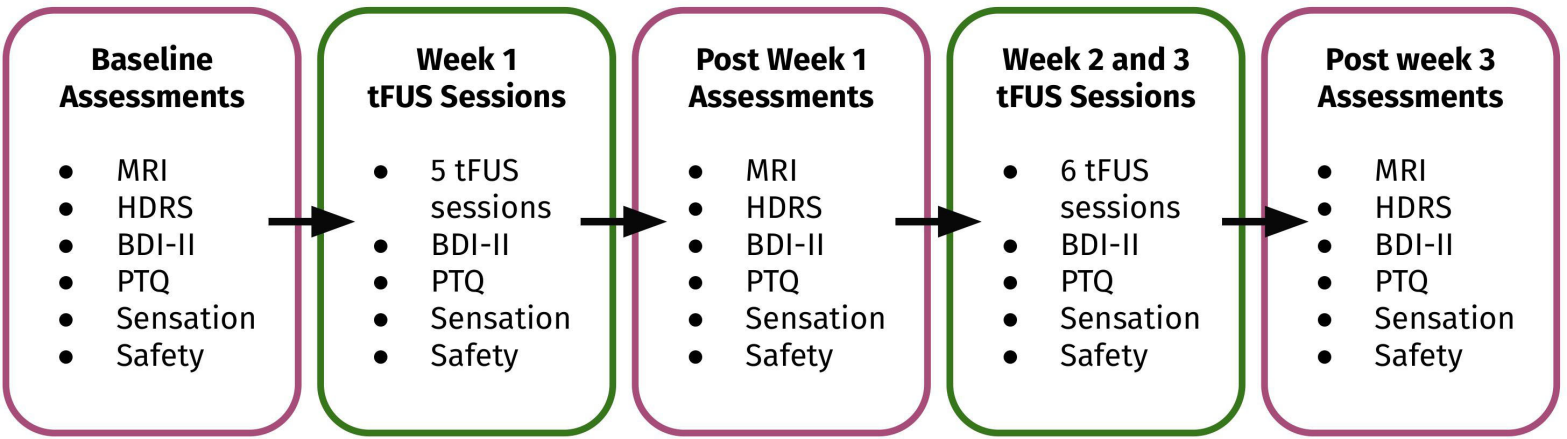
Summary of study procedures. Participants completed a baseline MRI scanning session, baseline symptom interview, and baseline self-report surveys before completing up to three weeks of transcranial-focused ultrasound (tFUS). Participants completed the same scanning, interview, and self-report surveys after completing one week of tFUS and after completing three weeks of tFUS (if applicable). Participants also completed self-report surveys and safety measures after each tFUS session.

This open-label trial of 20 participants involved five to eleven tFUS sessions across one to three weeks, with fMRI assessments at baseline, after week 1 of tFUS, and after week 3 of tFUS. A sample of 20 participants was selected to identify whether tFUS could produce a large effect size. **Figure 1** provides an overview of the study procedures which are also described in more detail below. The Institutional Review Board of the University of Arizona approved the experimental protocol (IRB approval number: STUDY00002019). All participants signed informed consent before participation. Clinical Trial Registration number: 019782-00001, https://clinicaltrials.gov/study/NCT06320028 identifier, NCT06320028.

### Inclusion criteria

**Figure 2:**
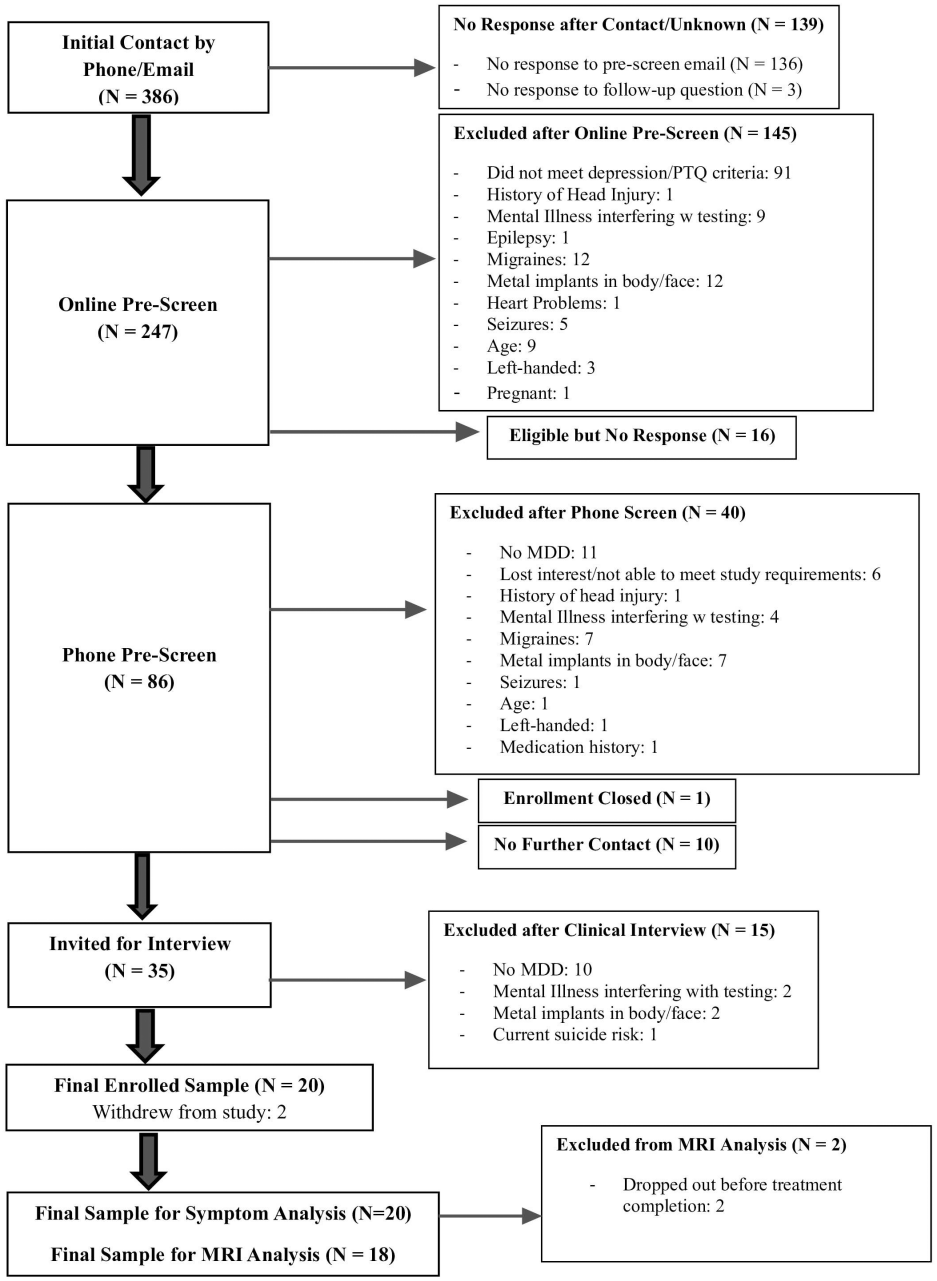
CONSORT diagram.

Individuals enrolled in the study met criteria for a current major depressive episode, diagnosed using the structured clinical interview for DSM-5 (SCID-5) (18), and had high levels of RNT as operationalized by a total score on the Perseverative Thinking Questionnaire (PTQ) (19) above 37 points (75^th^ percentile). To be included in the study, participants met the following additional criteria: age 18 - 50, right-handed, English-speaking, an absence of any of the following: history of neurological or psychotic symptoms, history of head injury with loss of consciousness, uncorrected vision and/or hearing impairment that would interfere with study procedures, current or history of brain illness likely to interfere with testing, current substance and/or alcohol dependence, a diagnosed sleep disorder, history of epilepsy and/or diagnosed migraines, metal implants in upper body (e.g., piercings, permanent dental retainer), history of cardiac problems influencing brain function (e.g., atrial fibrillation), current pregnancy, and current active suicidality necessitating immediate intervention. Prior resistance to psychopharmacological treatment was not a requirement for eligibility. Participants were instructed to continue current medications and psychological treatment as prescribed, as well as abstain from beginning any new medications and psychological treatment, for the duration of the trial. Twenty adults were enrolled in the study, with eighteen participants completing the full treatment protocol. Two participants withdrew from the study before completing their treatment protocol (CONSORT Diagram; **Figure 2**).

### tFUS target selection

The left amPFC (MNI coordinates: -5, 45, -3), part of a major hub of the DMN, was the chosen target due to its high degree of betweenness-centrality with other regions within the DMN (1). Left lateralization was chosen based on previous literature identifying the contribution left-lateralized frontal systems play in self-referential processing and mood (20–22).

### tFUS delivery

Each tFUS session consisted of a ten-minute delivery of the tFUS protocol, and each participant completed up to eleven sessions within a 3-week period. In week 1, participants completed five sessions within a seven-day period. If early remission criteria (described below) were not met after completion of week 1, participants completed two more weeks of tFUS, with each week involving three sessions within a seven-day period (six total additional sessions). During the ultrasound sessions, participants were instructed to sit quietly with eyes open, letting their thoughts come and go, a common approach used in resting-state fMRI literature (23, 24). Participants sat quietly for an additional 20 minutes, with eyes either open or closed, after tFUS delivery.

**Figure 3:**
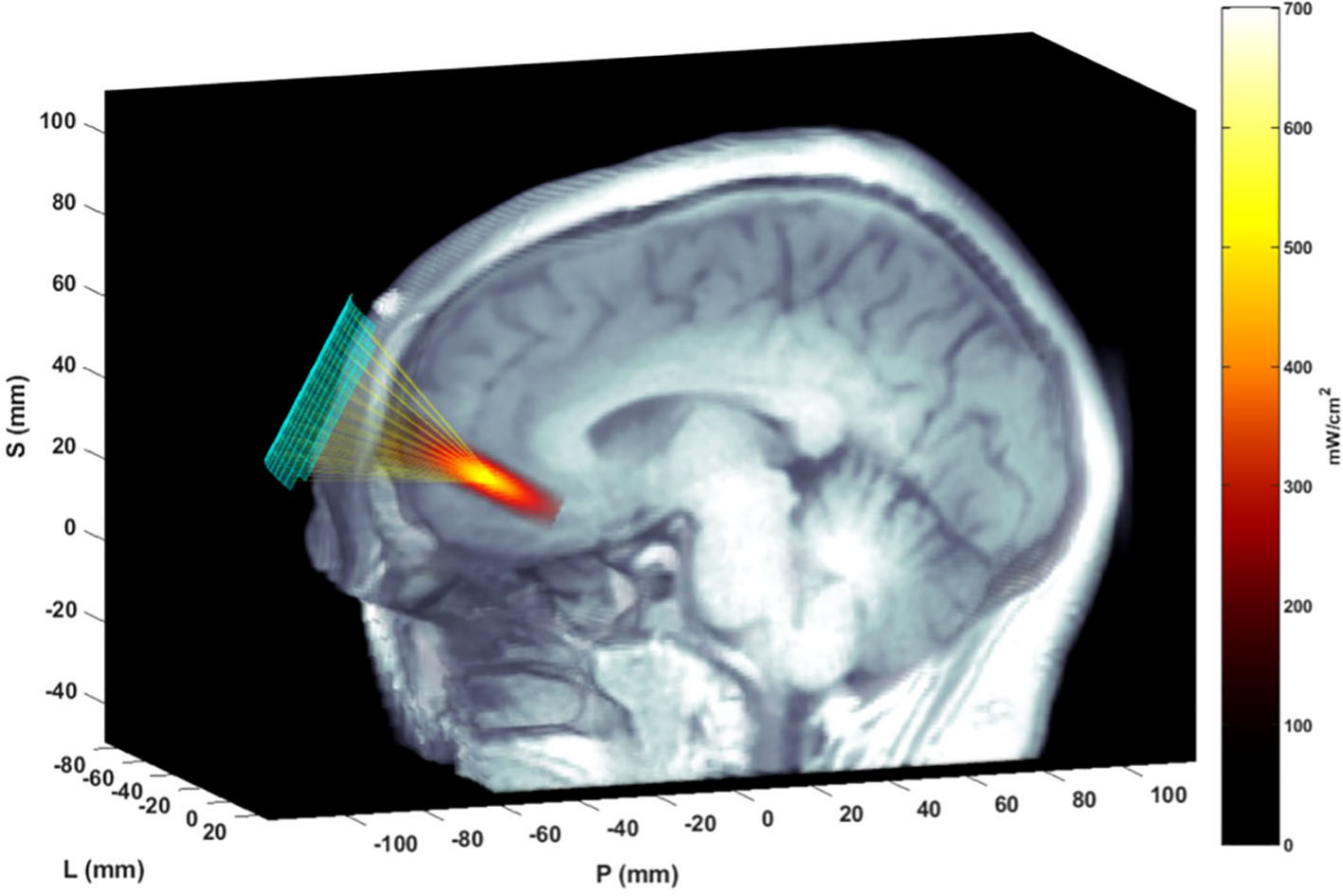
Simulation of ultrasound delivery to the amPFC using k-Wave modeling. A matrix array transducer was coupled to the participant’s forehead to transmit focused ultrasound through the skull to the intended target. The transducer’s placement was registered with the Localite TMSNavigator Neuronavigation system (Localite GmbH, Bonn, Germany). The modeled focal spot, calculated from simulated time delays via the k-Wave ultrasound toolbox, was superimposed on the MRI scan to illustrate the spatial-peak, temporal-average spatial distribution of acoustic intensity at the target site. This figure originally appeared in Schachtner et al. (13) and is reproduced here under the terms of the Creative Commons CC BY license.

The tFUS device was a custom Neuromodulation system (25) with a 128-element array. The ultrasound beam was steerable with the following parameters: acoustic frequency = 400 kHz, pulse duration = 5 ms, pulse repetition rate (PRR) = 10 Hz, a maximal spatial peak/temporal average acoustic intensity = 670 mW/cm^2,^ peak negative pressure 820 kPa. A custom-designed headset created by Openwater was used to secure the probe to the participants’ foreheads and to register its position relative to the target based on each participant’s structural MRI using Localite Neuronavigation Software (26) adapted for the tFUS device. This procedure afforded the ability to create a personalized treatment plan for each participant based on their specific left amPFC position. Precise targeting was achieved using electronic steering within the safety parameters for tFUS exposure (25), as well as a five sub-foci energy delivery approach within 5mm from each other, centered on the amPFC target. The general k-Wave modeling approach is detailed in Bawiec et al., 2024 (25) and the specific k-Wave modeling (13) is detailed in Schachtner et al., 2025. **Figure 3**, from Schachtner et al. (2025), depicts the results of the k-Wave modeling with this steerable system and the left amPFC target.

### Depression assessments

Before engaging in the tFUS treatment protocol, after completing week 1 of tFUS, and after completing week 3 of tFUS (if applicable), participants completed self-report assessments and clinical interviews, which included: The Beck Depression Inventory – II (BDI-II) (27), the PTQ, the Columbia–Suicide Severity Rating Scale (CSSR-S) (28), and the Hamilton Depression Rating Scale (HDRS) (29). Additionally, after each tFUS session, the BDI-II and PTQ were administered to assess day-to-day changes in symptoms. After completing week 1 of tFUS, an assessment evaluated whether early remission was obtained. Early remission criteria included a BDI-II score of <13, a HDRS score of <8, and a PTQ score of <18. Participants continued for two more weeks of tFUS if they did not meet all three criteria. Once participants completed the entire tFUS protocol, remission was reassessed. Response was defined as a reduction of scores below 50% of baseline, an approach commonly used in intervention studies (30).

### MRI data acquisition

Participants completed MRI sessions before beginning ultrasound treatment, after week 1 of tFUS, and after week 3 of tFUS (if applicable). Each session included a T1-weighted structural scan, a PETRA scan (with short TE for assessing skull density), a twelve-minute BOLD resting-state fMRI scan, and a Susceptibility Weighted Image (SWI) scan. The PETRA and T1 scans were used for localization and targeting, and the SWI scans were reviewed by board-certified neurologists to assess the possibility of micro-hemorrhaging.

All scans were acquired on the Siemens Skyra 3-Tesla Scanner. T1-weighted anatomical scans were acquired for registration of the functional scans (MP-RAGE; TR = 2100 ms; TE = 2.33 ms; TI = 1100 ms; flip angle = 12; FOV was 256 mm) and for the fMRI analyses. Functional images were acquired using EPI gradient echo sequence (TR = 2000 ms; TE = 30 ms; flip angle = 70; FOV = 240 mm; acquisition voxel size 2 mm × 2 mm × 2 mm).

The PETRA scans were acquired with the following parameters: TR = 5 ms; TE = 0.07 ms; flip angle = 6; FOV = 240 mm; acquisition voxel size 0.9 mm × 0.9 mm × 0.9 mm. The SWI scans were acquired with the following parameters: TR = 28 ms; TE = 20 ms; flip angle = 15; FOV = 220 mm; acquisition voxel size 0.6 mm × 0.6 mm × 1.5 mm.

### Adverse events

To assess potential side effects of tFUS, a sensation questionnaire was administered after each tFUS session, which included potential side effects commonly reported in TMS interventions (i.e., itching, heat/burning, tingling, vibrating/pulsing, sound, tension, and pain). Before each subsequent ultrasound session, participants were asked whether they had experienced any adverse sensations or experiences since the last tFUS administration. If a participant reported a potential adverse event, the onset and duration were logged, the severity was assessed, and the relationship to the tFUS intervention was determined. As an objective measure of tFUS safety, SWI scans were reviewed by board-certified neuroradiologists for potential neuron and vasculature damage. Schachtner et al. (2025) (13) provides detailed descriptions of the adverse event protocol, results, and discussion of findings.

### MRI preprocessing

Structural MRI and resting-state functional MRI data were preprocessed using fMRIprep 23.0.2 (RRID: SCR_016216), a standardized preprocessing pipeline widely used among MRI researchers (31). Structural images underwent skull-stripping, segmentation, spatial normalization to Montreal Neurological Institute (MNI) space, and surface reconstruction. Functional images underwent susceptibility distortion correction, realignment, slice timing correction, co-registration, and spatial normalization to standard space. Preprocessed fMRIprep data were loaded into CONN toolbox 22.a (32) for additional preprocessing, including outlier detection, spatial smoothing, and denoising. For a detailed description of the fMRIprep and CONN toolbox preprocessing steps, please see the **Supplementary Materials**.

Scans were analyzed at baseline and end of treatment. For 17 out of 18 participants, end-of-treatment was defined as week 3. One participant met early remission criteria following week 1, which was used as their end-of-treatment scan. Two participants withdrew from the study before completing weeks 2–3 of tFUS and final assessments and scans, which excluded them from the MRI analysis. Eighteen out of twenty participants were included in the fMRI analysis. 

### Statistical analysis

#### Depression symptoms and RNT

Multi-level models (MLM) were used to assess changes in depression symptoms (BDI-II and HDRS) and RNT (PTQ) over the course of treatment for all 20 participants enrolled in the study. Additionally, two linear regressions were employed to assess the relationship between change in self-report depression symptoms and change in RNT, as well as the relationship between change in clinical interview depression rating and change in RNT for all 20 participants enrolled in the study. For a detailed description of the statistical specifications, please see Schachtner et al. (2025) (13).

#### DMN connectivity changes

ROI-to-ROI connectivity (RRC) matrices were estimated characterizing the functional connectivity between each pair of regions among six ROIs that comprise the major hubs of subsystem A of the DMN: leftPFC (dorsal), leftPFC (medial), leftPCC, rightPFC (dorsal), rightPFC (medial), and rightPCC using the Schaefer 17-network, 100-parcellation atlas (33). These six ROIs were selected to represent Subsystem A (1) of the DMN to assess specific within-network changes. Functional connectivity strength was represented by Fisher-transformed bivariate correlation coefficients from a general linear model (weighted-GLM) (34), estimated separately for each pair of ROIs, characterizing the association between their BOLD signal timeseries. Individual scans were weighted by a boxcar signal characterizing each individual session convolved with an SPM canonical hemodynamic response function and rectified.

Group-level analyses were performed using a General Linear Model (GLM) (34). For each connection between the six DMN ROIs, a separate GLM was estimated, with first-level connectivity measures for each connection as the dependent variable, and time (end of treatment (-1) vs baseline (1)) as the independent variable. Connection-level hypotheses were evaluated using multivariate parametric statistics with random-effects across participants and sample covariance estimation across multiple measurements. Inferences were performed at the level of individual clusters (groups of contiguous connections). Cluster-level inferences were based on nonparametric statistics from the standard Spatial Pairwise Clustering analyses (SPC) (35) setting in CONN toolbox, with 1000 residual-randomization iterations, and ROIs sorted using optimal leaf ordering based on ROI-to-ROI anatomical proximity and functional similarity metrics (34, 36). Results were thresholded using a combination of a cluster-forming p < 0.01 connection-level threshold and a familywise corrected p-FDR < 0.05 cluster-mass threshold (37).

### Relationship between change in symptoms and connectivity

Baseline and end-of-treatment connectivity values for the significant ROI-to-ROI pair (i.e., leftPFC-leftPCC) were extracted to employ two separate linear regression models examining the relationship between change in depression symptoms and change in leftPFC–leftPCC connectivity, and the relationship between change in RNT and change in leftPFC–leftPCC connectivity.

## Results

### Participant demographics

The sample was predominantly female (75%) and a majority were employed either part time (45%), full-time (15%), or were students (15%). The rest of the sample reported unemployment (25%). Thirty percent of the participants did not provide race and ethnicity information, and 45% identified as White, 10% as Black, 5% as Chinese, 5% as Indian, and 5% as Middle Eastern (**Table 1**).

**Table 1:** Participant demographics.

Demographics		N = 20
Age, Mean (SD)		30.35 (10.04)
Gender (F/M/Other), Count		14/4/1
Years of education, Mean (SD)		13.83 (1.93)
Race, %		
	White	45
	Black	10
	Chinese	5
	Middle Eastern	5
	Indian	5
	Unknown	30
Ethnicity, %		
	Hispanic	0
	Non-Hispanic	70
	Unknown	30
Employment, %		
	Full-time	15
	Student	15
	Part-time	45
	Unemployed	25
Baseline BDI-II, Mean (SD)		38.85 (9.34)
Baseline PTQ, Mean (SD)		44.35 (6.24)
Baseline HDRS, Mean (SD)		19.90 (6.34)
Depression onset (Early/Teen/Adult), %		55/25/20
Comorbidities, %		
	Anxiety and Stress-related Disorder	85
	Trauma-related Disorder	15
	Attention Deficit Hyperactivity Disorder	35
	Eating Disorder	5
	Persistent Depressive Disorder	55
History of Suicidal Ideation (None/Passive/Active), %		10/30/60
Hospitalization History for Mental Health (Any), %		35
History of Suicide Attempts (None/One/Multiple), %		70/15/15
Past Treatment, %		90
	Medication	75
	Psychotherapy	60
Current Treatment, %		90
	Medication, %	50
	Psychotherapy, %	20
Current Medication Type, %		
	SSRI (Luxov, Prozac, Sertraline)	15
	SARI (Trazadone)	5
	NDRI (Wellbutrin)	10
	Anti-convulsant (Gabapentin, Lamotrigine)	15
	Beta-Blockers (Propranolol)	5
	CNS stimulant (Adderall, Vyvanse)	10
	Sedative (propofol)	5
	Anti-hypertensives (Clonidine)	10

### Past and current treatment history

This sample was not uniformly treatment-resistant because resistance to prior psychopharmacological treatment (e.g., ≥2 adequate antidepressant trials ± psychotherapy) was not required for eligibility; however, ninety percent of participants reported past treatment attempts, with 75% attempting medication treatment and 60% attempting psychotherapy treatment. Ninety percent of participants were engaged with some form of treatment during the study, either medication (50%) or psychotherapy (20%). Despite past and current treatment history, all participants included in the study were diagnosed with current MDD.

### Safety

Participants’ reports of aversive sensations on the sensation questionnaire (itching, heat/burning, tingling, vibrating/pulsing, sound, tension, and pain) were all below a mean of 1.6 and the modal and median report was 0 (no sensation). Those who reported pain and tension from the treatment stated that these sensations were from the headset and not the tFUS signal delivery, itself. No serious adverse events were reported by any of the participants. One participant reported a temporary increase in suicidal ideation during the post-week 3 assessment that was unrelated to the study procedures.^1^ Two out of twenty participants withdrew from the treatment protocol after completing week 1, when instructed to complete two more weeks of tFUS, because they did not experience a subjective benefit from the treatment. Schachtner et al. (2025) provides a detailed discussion on these results (13).

Board-certified neuroradiologists read the SWI scans that were collected at baseline and end of treatment to assess vascular microhemorrhaging as an objective measure of tFUS safety. Across all 20 enrolled participants and scans, there were no microhemorrhages identified that could have resulted from tFUS delivery. Three out of 20 participants had baseline scans that showed pre-existing, non-specific white matter hyperintensities that did not change over the course of tFUS delivery.

### tFUS delivery

**Table 2** provides the per-participant mean (sd) targeting accuracy, skull-related pressure loss, and acoustic exposure metrics. Navigation error represents the distance (mm) between the intended and simulated target location. Estimated focal pressure through the skull was derived from acoustic simulations and compared to the intended water-focus pressure (0.82 MPa) to calculate percent loss and transmission values. Spatial-peak temporal-average intensity (Ispta; mW/cm²) was computed using the corrected 5% duty cycle, and acoustic dose (J/cm²) was calculated as Ispta multiplied by the 10-minute ultrasound delivery. Targeting accuracy was generally very good in the XY plane, but less so in the Z dimension as would be expected by the skull aberration of the beam, which tends to shorten the focal length. This is also reflected in the greatest loss of energy through the skull for the greatest deviation in the Z dimension, both reflecting a greater impact of the skull for these participants.

**Table 2:** Per-participant mean (sd) targeting accuracy, skull-related pressure loss, and acoustic exposure metrics.

Participant	Targeting accuracy XY (mm)	Targeting accuracy Z (mm)	Focal pressure (MPa)	% Loss	Acoustic power (W/cm^2)	Acoustic dose (J/cm^2)
01	NA	NA	NA	NA	NA	NA
04	2.52 (0.75)	6.18 (2.52)	0.37 (0.09)	0.55 (0.11)	218.28 (93.52)	130.97 (56.11)
05	1.56 (0.80)	10.24 (1.49)	0.42 (0.03)	0.49 (0.04)	269.49 (43.98)	161.69 (26.39)
06	1.04 (0.40)	4.01 (0.82)	0.53 (0.03)	0.36 (0.03)	428.16 (41.61)	256.90 (24.97)
07	2.07 (0.38)	6.58 (2.22)	0.46 (0.05)	0.44 (0.06)	332.62 (63.81)	199.57 (38.28)
08	3.62 (0.94)	17.15 (8.22)	0.17 (0.04)	0.80 (0.05)	44.90 (22.62)	26.94 (13.57)
09	0.61 (0.35)	5.35 (1.49)	0.58 (0.02)	0.30 (0.02)	514.79 (29.02)	308.87 (17.41)
10	1.71 (0.98)	9.63 (3.54)	0.41 (0.07)	0.50 (0.09)	265.01 (91.73)	159.00 (55.04)
11	1.89 (0.44)	7.71 (2.28)	0.32 (0.00)	0.61 (0.00)	158.38 (3.11)	95.03 (1.87)
12	1.87 (0.59)	8.53 (4.85)	0.31 (0.05)	0.62 (0.06)	150.81 (48.27)	90.49 (28.96)
13	1.95 (1.28)	11.04 (6.47)	0.33 (0.08)	0.60 (0.10)	175.31 (74.10)	105.18 (44.46)
14	1.79 (1.07)	7.61 (1.46)	0.31 (0.08)	0.63 (0.09)	152.75 (78.20)	91.65 (46.92)
15	0.76 (0.40)	4.31 (0.81)	0.47 (0.01)	0.43 (0.02)	335.38 (21.36)	201.23 (12.82)
16	1.45 (0.48)	1.41 (1.82)	0.53 (0.02)	0.36 (0.02)	432.50 (26.60)	259.50 (15.96)
17	1.29 (0.32)	5.62 (0.75)	0.59 (0.02)	0.28 (0.02)	540.47 (34.05)	324.28 (20.43)
18	1.34 (0.41)	6.03 (1.45)	0.36 (0.03)	0.56 (0.04)	199.01 (37.24)	119.40 (22.34)
19	0.65 (0.27)	3.70 (1.12)	0.53 (0.01)	0.36 (0.01)	429.89 (18.76)	257.93 (11.26)
20	1.31 (0.39)	10.73 (3.62)	0.28 (0.05)	0.66 (0.06)	127.01 (44.69)	76.20 (26.82)

Data were available for 17 out of 18 participants included in the MRI analysis. Participants 02 and 03 withdrew from the study and were not included in the MRI analysis. Means and SDs are derived from all available tFUS sessions for each participant.

### Depression symptoms and RNT

**Figure 4:**
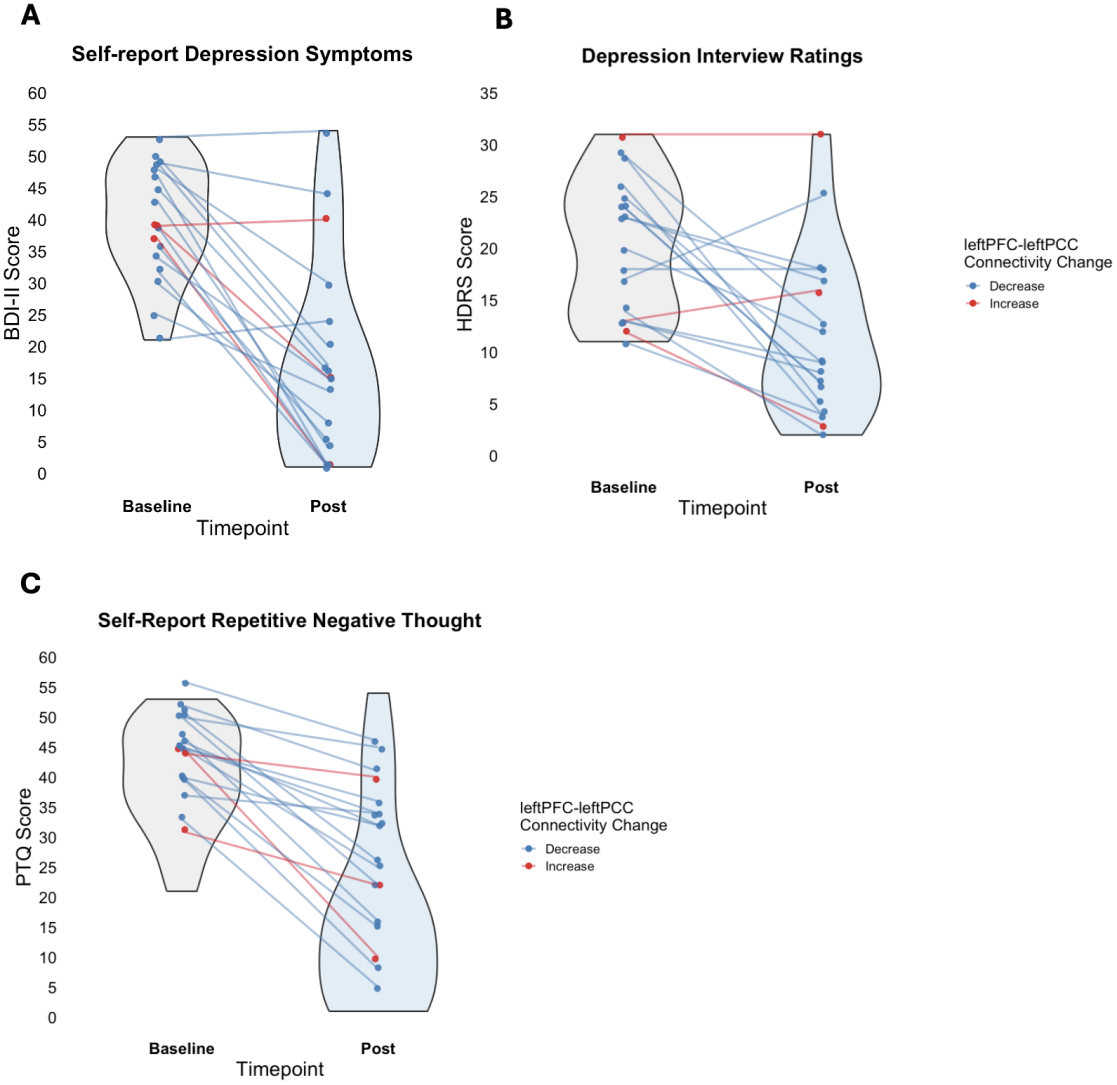
Effect of transcranial-focused ultrasound treatment on depression symptoms and repetitive negative thought from baseline to end-of-treatment for the 18 participants included in the MRI analysis. **(A)** Significant decrease in Beck-Depression Inventory – II (BDI-II), **(B)** Significant decrease in Hamilton Depression Rating Scale (HDRS), and **(C)** Significant decrease in Perseverative Thinking Questionnaire (PTQ). Blue lines indicate decreased PFC-PCC connectivity and red lines indicate increased PFC-PCC connectivity. For the figure summarizing the MLM findings for all 20 participants reported in the results section, please see Schachtner et al., 2025 (13).

For the results reported in Schachtner et al. (2025) (13), all 20 individuals were included in the analyses for the purpose of assessing the full intent-to-treat sample, and there was a significant decrease in depression symptoms and RNT after treatment. Beck depression inventory-II and HDRS total scores, indices of depression symptoms, decreased by 10.9 (p < 0.001, CI (95%) = -13.55, -8.15) and 4.2 (p < 0.001, CI (95%) = -5.85, -2.62), respectively, per time point (baseline to week 1, baseline to week 3). Perseverative thinking questionnaire total scores, an index of RNT, decreased by 8.4 (p <0.001, CI (95%) = -10.55, -6.03) per time point. Additionally, there was a significant and positive relationship between change in depression symptoms and change in RNT, where those with greater decreases in self-report (*R*^2^ = 0.67, F = 36.84 (1, 18), p < 0.001, slope CI (95%)= 0.76, 1.57) and clinical interview (*R*^2^ = 0.37, F = 10.59 (1, 18), p = 0.004, slope CI (95%)= 0.17, 0.79) depression symptoms experienced a greater decrease in RNT. **Figures 4a–c** show change in symptom severity for BDI-II, HDRS, and PTQ, respectively, from baseline to end-of-treatment for the 18 participants included in the MRI analysis to illustrate how the participants included in the present paper changed in symptom severity, while also illustrating who decreased or increased in leftPFC-leftPCC connectivity.

### DMN connectivity

**Figure 5:**
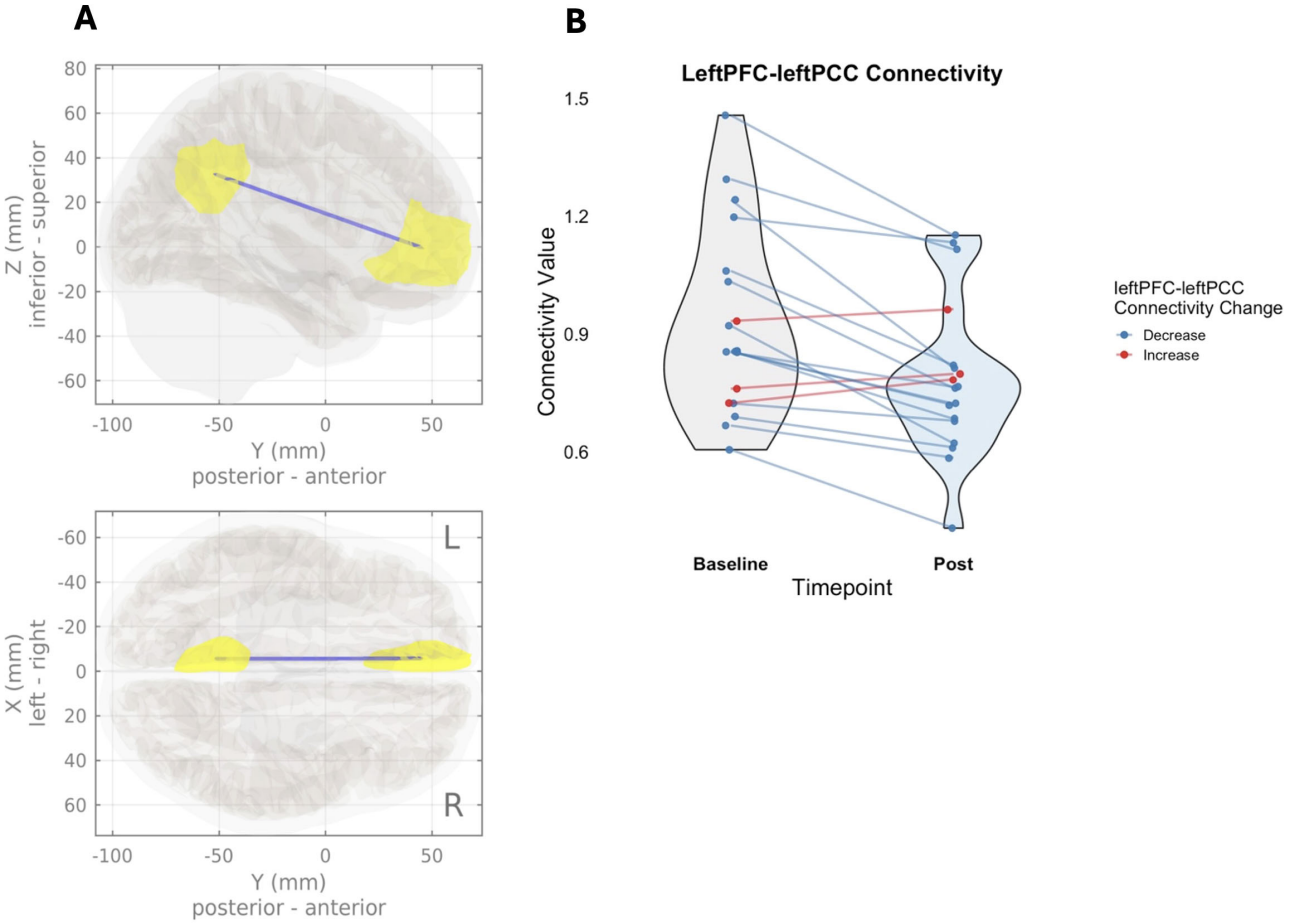
ROI-to-ROI default mode network (DMN) functional connectivity changes from baseline to end of treatment. **(A)** Sagittal and coronal views of a significant decrease in DMN ROI-to-ROI functional connectivity, between the left posterior cingulate cortex (posterior) and left medial prefrontal cortex (anterior). Decreased connectivity is represented by the blue line, with the affected ROIs highlighted in yellow. **(B)** Violin plot of significant change in ROI-to-ROI DMN connectivity between the left posterior cingulate cortex and left medial prefrontal cortex, with individual change plots for each of the 18 participants overlayed. Blue lines represent individuals who decreased in connectivity and red lines represent individuals who increased in connectivity.

There was a significant decrease in connectivity within the DMN cluster (p_fdr_ = 0.017, mass = 43.13). Within the cluster, there was a significant decrease in connectivity within subsystem A of the DMN, between the leftPFC and leftPCC (p_fdr_ = 0.003, T(17) = -4.64), with 15 of the 18 participants showing this decreased connectivity pattern (**Figure 5**). **Table 3** provides summary statistics for the change observed in leftPFC-leftPCC connectivity and change in primary clinical symptoms, from baseline to end-of treatment. The summary statistics were calculated using only the 18 participants included in the MRI analysis.

**Table 3:** Summary statistics for leftPFC-leftPCC connectivity, Beck Depression Inventory-II (BDI-II), Hamilton Depression Rating Scale (HDRS), and Perseverative Thinking Questionnaire (PTQ) from baseline to end of treatment for the 18 participants included in the MRI analysis.

Outcome	Baseline mean (sd)	End of treatment mean (sd)	Cohen’s D	Effect size CI (95%)
leftPFC-leftPCC Connectivity	0.93 (0.24)	0.79 (0.20)	-1.90	-2.60, -1.21
BDI-II	39.78 (9.05)	17.17 (15.90)	-1.37	-2.21, -0.54
HDRS	20.28 (6.57)	11.56 (7.98)	-0.84	-1.54, -0.14
PTQ	44.28 (6.56)	27.17 (12.68)	-1.314	-2.54, -0.74

### DMN connectivity change and symptom change

There was no significant relationship between change in DMN connectivity and change in self-report depression symptoms (BDI-II; *R*^2^ = -0.02, F = 0.66 (1, 16), p = 0.43, slope CI (95%) = -21.20, 47.64) or clinical interview depression rating (HDRS; (*R*^2^ = -0.04, F = 0.41 (1, 16), p = 0.53, slope CI (95%) = -41.34, 77.33), nor was there a significant relationship between change in DMN connectivity and change in RNT (PTQ; (*R*^2^ = -0.06, F = 0.07 (1, 16), p = 0.79, slope CI (95%) = -37.68, 48.85). **Table 4** provides a zero-order, simple correlation matrix to illustrate the relationship between change in depression symptoms, RNT, and DMN connectivity in the 18 participants included in the MRI analysis.

**Table 4:** Zero-order correlation matrix illustrating relationship between change in PFC – PCC connectivity and change in the Beck Depression Inventory-II (BDI-II), Hamilton Depression Rating Scale (HDRS), and Perseverative Thinking Questionnaire (PTQ) for the 18 participants included in the MRI analysis.

Outcome	Δ PFC -PCC connectivity	Δ HDRS	Δ BDI-II	Δ PTQ
Δ centerPFC-centerPCC Connectivity	1.00 (p = 0.00)	0.20 (p = 1.00)	0.16 (p = 1.00)	0.07 (p = 1.00)
Δ HDRS	0.20 (p = 0.43)	1.00 (p = 0.00)	0.63 (p = 0.02)	0.58 (p = 0.04)
Δ BDI-II	0.16 (p = 0.53)	0.63 (p = 0.01)	1.00 (p = 0.00)	0.81 (p = 0.00)
Δ PTQ	0.07 (p = 0.79)	0.58 (p = 0.01)	0.81 (p = 0.00)	1.00 (p = 0.00)

Connectivity change was calculated as end-of-treatment minus baseline. Symptom change was calculated baseline minus end-of-treatment.

## Discussion

### DMN connectivity changes

There was a significant decrease in functional connectivity within a major hub of the DMN, between the leftPFC and leftPCC, after tFUS treatment in an MDD sample. Our findings align with previous studies examining the impact of various depression treatments on DMN connectivity. For example, Posner et al. (2013) found that, after a 10-week antidepressant regime, those with dysthymic disorder experienced a decrease in DMN connectivity (38). Research using TMS has also reported reductions in DMN connectivity alongside improvements in depressive symptoms, underscoring the broader neuromodulatory and antidepressive potential of brain stimulation techniques targeting this specific network (39).

Riis and colleagues have also recently demonstrated the promise of tFUS treatment for depression by targeting the limbic system, which functions aberrantly in depression (40), and found that targeting the subcallosal cingulate cortex (SCC) led to decreases in brain activity within this target and also decreases in depression symptoms (41, 42). The cortico-limbic theory of depression postulates that depression manifests from the dysfunction between cortical (e.g., PFC) and limbic regions (e.g., SCC) (40) and may explain why dissimilar targeting, compared to the present study, resulted in similar symptom reduction. Future work examining the impact of tFUS on connectivity between brain networks will be an important next step.

Additionally, Riis and colleagues analyzed their MRI data on an individual patient level, with only seven out of twelve participants experiencing a significant alteration in SCC brain activity after treatment (41). In the present study, although there was a significant decrease in DMN connectivity on a group level, three out of 18 participants showed, descriptively, an increase in connectivity after treatment (**Figure 5**). Future work should explore differential targeting to understand whether other DMN targets may be optimal, and whether targeting based on the individual’s baseline characteristics, such as brain connectivity patterns and symptom severity, might also portend a larger response.

### DMN connectivity, depression symptoms, and RNT

There was no significant relationship between change in depression symptoms and change in leftPFC- leftPCC connectivity, nor was there a significant relationship between change in RNT and change in leftPFC- leftPCC connectivity. Literature suggests that the DMN plays an integral role in the development and maintenance of depression and RNT; however, these studies have primarily compared patients versus controls at baseline, wherein those who are depressed present with DMN hyperconnectivity (43) and those who experience greater RNT experience greater DMN connectivity (44).

In contrast to the consistency in DMN connectivity findings comparing depressed to non-depressed samples, the relationship between symptom change and connectivity change over the course of treatment within a depressed sample has a pattern of predominantly null results in the literature. A review summarizing the effects of various depression treatments (e.g., medication, psychotherapy, ECT, and TMS) on brain connectivity highlighted several studies that assessed DMN connectivity (45). Eight studies found significant improvement in depression symptoms alongside significant alterations in DMN connectivity, both within the DMN and between other networks (e.g., CEN), among which five studies specifically assessed the relationship between symptom change and DMN connectivity change. Out of those five studies, only two observed a significant relationship between symptom changes and connectivity changes, with only one study specifically suggesting that increased connectivity between the dorsomedial PFC and thalamus correlated with depression symptom improvement (45). It is important to note that these studies did not have a control group. Sample sizes also widely varied across studies, with Ns ranging from 8 to 41. One study with a relatively large N of 41 and a control group of 25 did not find an association between symptom change and connectivity change.

These findings highlight the difficulty in relating symptom change to connectivity change and raise important methodological and theoretical questions. First, heterogeneity in clinical presentation (e.g. symptom severity and which symptoms are present) and variability in the degree of aberrant DMN connectivity at baseline may play important roles in this relationship. Understanding how these individual differences impact the relationship between symptom and DMN connectivity change may provide the opportunity to identify, in an individualized precision-medicine framework, those that may be the best candidates for specific treatments or specific targets using tFUS. Second, these results raise the possibility that tFUS modulating DMN connectivity may be necessary to observe symptom improvement, but not sufficient on its own. This view would recognize tFUS to the DMN as a catalyst for change, with other factors determining whether such change occurs and its magnitude. There is currently no research that assesses the synergistic effects of tFUS and other therapeutic techniques (e.g., cognitive behavioral therapy) (46), and this may be needed to establish a clear relationship between changes in connectivity and changes in symptoms.

### Limitations and future directions

The present study provides important, initial evidence for the promise of tFUS as a targeted intervention for MDD that directly modulates a brain network implicated in this disorder, the DMN. The current sample was not uniformly treatment resistant; however, these findings provide a foundation for exploring whether tFUS is a non-invasive, viable treatment option for those who are uniformly resistant to currently available treatment options. A limitation of this open-label study was the lack of a control group and small sample size, which commonly occurs in MRI studies (47). To rigorously assess whether there is a causal relationship between tFUS delivery and changes in DMN connectivity, a randomized controlled trial with active and sham tFUS delivery is needed to control for nonspecific therapeutic factors. Future work requiring a larger sample size and increased power will aim to further explore the relationship between change in DMN connectivity and change in depression symptoms and RNT, as well as the relationship between baseline connectivity and change in symptoms. Additionally, this sample was extremely heterogenous, which can lead to difficulty in identifying clear, consistent neural mechanisms of depression across individuals (48); however, the present study’s strength is that it represents a real-world sample, as many individuals with MDD have comorbid diagnoses (49).

Energy delivery to the target was relatively precise within the XY plane, with some distortion related to skull aberration in the Z dimension. Future studies can correct for skull aberration to provide a more consistent focal delivery of the intended energy. Diffusion Tensor Imaging (DTI) is an MRI approach used to identify the integrity of structural connections in the brain (50), and has been used to guide neuromodulation via DBS by verifying the structural integrity between the tFUS target and sub-regions implicated in the network of interest (e.g., DMN) (51). Future work should consider the use of DTI when planning treatment protocols to personalize treatment to the individuals structural and functional connectivity. Despite these limitations, the findings provide a strong foundation for implementing tFUS as a treatment for depression with pronounced and rapid anti-depressant effects, suggesting the promise of a randomized clinical trial to establish efficacy and explore the causal role of DMN connectivity in depression.

## Data Availability

The raw data supporting the conclusions of this article will be made available by the authors, without undue reservation.

## Footnotes

1 Despite experiencing a transient increase in suicidal ideation, this participant experienced a decrease in DMN connectivity.
