# Supplementary material for: The effect of non-invasive transcranial focused ultrasound for depression on the default mode network: an open-label pilot trial
Source: Front Psychiatry. 2026 Jan 20;16:1722575. doi: 10.3389/fpsyt.2025.1722575 (PMC12865718; doi:10.3389/fpsyt.2025.1722575)
Supplement: Supplementary file 1 [file Table1.docx]

**Supplemental Material**

**fMRIprep Anatomical MRI Preprocessing**

The following preprocessing was completed for each subject’s dataset. One T1-weighted (T1w) image was found within the input BIDS dataset. The T1-weighted (T1w) image was corrected for intensity non-uniformity (INU) with N4BiasFieldCorrection (4) distributed with ANTS 2.3.3 (RRID:SCR_004757) (5), and used as T1w-reference throughout the workflow. The T1w-reference was then skull-stripped with a Nipype implementation of the antsBrainExtraction.sh workflow (from ANTs), using OASIS30ANTs as target template. Brain tissue segmentation of cerebrospinal fluid (CSF), white-matter (WM) and gray-matter (GM) was performed on the brain-extracted T1w using fast (FSL, RRID:SCR_002823) (6). Brain surfaces were reconstructed using recon-all (FreeSurfer 7.3.2, RRID:SCR_001847) (7), and the brain mask estimated previously was refined with a custom variation of the method to reconcile ANTs-derived and FreeSurfer-derived segmentations of the cortical gray-matter of Mindboggle (RRID:SCR_002438) (8). Grayordinate “dscalar” files (9) containing 91k samples were also generated using the highest-resolution fsaverage as an intermediate standardized surface space. Volume-based spatial normalization to two standard spaces (MNI152NLin6Asym, MNI152NLin2009cAsym) was performed through nonlinear registration with ants Registration (ANTs) using brain-extracted versions of both T1w reference and the T1w template. The following templates were selected for spatial normalization and accessed with TemplateFlow (23.0.0) (10) FSL’s MNI ICBM 152 non-linear 6th Generation Asymmetric Average Brain Stereotaxic Registration Model (RRID:SCR_002823; TemplateFlow ID: MNI152NLin6Asym) (11), ICBM 152 Nonlinear Asymmetrical template version 2009c (RRID:SCR_008796; TemplateFlow ID: MNI152NLin2009cAsym) (12).

**fMRIprep Functional MRI preprocessing**

For each of the two BOLD runs found per subject (i.e., baseline and end of treatment scans), the following preprocessing was performed. First, a reference volume and its skull-stripped version were generated using a custom methodology of fMRIPrep. Head-motion parameters with respect to the BOLD reference (transformation matrices, and six corresponding rotation and translation parameters) are estimated before any spatiotemporal filtering using mcflirt (FSL) (13). The BOLD time-series (including slice-timing correction when applied) were resampled onto their original, native space by applying the transforms to correct for head-motion. These resampled BOLD time-series will be referred to as preprocessed BOLD in original space, or just preprocessed BOLD. The BOLD reference was then co-registered to the T1w reference using bbregister (FreeSurfer) which implements boundary-based registration (14). Co-registration was configured with six degrees of freedom. Several confounding time-series were also calculated and stored by fMRIPrep (e.g., motion parameters, global signals, CompCor components), however, these were not used in the denoising step in CONN Toolbox. Instead, this step was carried out exclusively using CONN Toolbox, along with subsequent spatial smoothing and denoising steps.

The BOLD time-series were resampled into standard space, generating a preprocessed BOLD run in MNI152NLin6Asym space. First, a reference volume and its skull-stripped version were generated using a custom methodology of fMRIPrep. The BOLD time-series were resampled onto the following surfaces (FreeSurfer reconstruction nomenclature): fsnative, fsaverage. The BOLD time-series were resampled onto the left/right-symmetric template “fsLR” (9). Grayordinates files (9) containing 91k samples were also generated using the highest-resolution fsaverage as intermediate standardized surface space. All resamplings can be performed with a single interpolation step by composing all the pertinent transformations (i.e. head-motion transform matrices, susceptibility distortion correction when available, and co-registrations to anatomical and output spaces). Gridded (volumetric) resamplings were performed using antsApplyTransforms (ANTs), configured with Lanczos interpolation to minimize the smoothing effects of other kernels (15). Non-gridded (surface) resamplings were performed using mri_vol2surf (FreeSurfer). Many internal operations of *fMRIPrep* use *Nilearn* 0.9.1(RRID:SCR_001362) (16), mostly within the functional processing workflow. For more details of the pipeline, see Power et. al. (2017) (17) and Brett et. al. (2001) (18).

**CONN Toolbox Preprocessing**

Potential outlier scans were identified using ART (19) to identify acquisitions with framewise displacement above 0.9 mm or global BOLD signal changes above 5 standard deviations (20,21), and a reference BOLD image was computed for each subject by averaging all scans excluding outliers. Finally, functional data were smoothed using spatial convolution with a Gaussian kernel of 6 mm full width half maximum (FWHM). In addition, functional data were denoised using a standard denoising pipeline (22) including the regression of potential confounding effects characterized by white matter timeseries (5 CompCor noise components), CSF timeseries (5 CompCor noise components), motion parameters and their first order derivatives (12 factors) (23), outlier scans (below 10 factors) (21), effect of time and the first order derivatives (4 factors), and linear trends (2 factors) within each functional run, followed by bandpass frequency filtering of the BOLD timeseries (24) between 0.008 Hz and 0.09 Hz. CompCor (25,26) noise components within white matter and CSF were estimated by computing the average BOLD signal as well as the largest principal components orthogonal to the BOLD average, motion parameters, and outlier scans within each subject's eroded segmentation masks. From the number of noise terms included in this denoising strategy, the effective degrees of freedom of the BOLD signal after denoising were estimated to range from 213.9 to 217.8 (average 217.1) across all subjects (20).

**fMRIprep Copyright Waiver**

The above boilerplate text was automatically generated by fMRIPrep. It is released under the CC0 license (27). Minor edits to the text were made for clarity, including tense adjustments, incorporating study-specific information (e.g., “baseline” and “end of treatment”), and reformatting citations to comply with the journal requirements. Additionally, the authors added the statement about outlier detection and confound regression being conducted in CONN Toolbox. The authors removed the irrelevant information from the boilerplate related to outlier detection.

**Supplemental References**

1. Esteban O, Markiewicz CJ, Blair RW, Moodie CA, Isik AI, Erramuzpe A, *et al.* (2019): fMRIPrep: a robust preprocessing pipeline for functional MRI. *Nat Methods* 16: 111–116.

2. Gorgolewski K, Burns CD, Madison C, Clark D, Halchenko YO, Waskom ML, Ghosh SS (2011): Nipype: a flexible, lightweight and extensible neuroimaging data processing framework in python. *Front Neuroinform* 5: 13.

3. Gorgolewski KJ, Esteban O, Ellis DG, Notter MP, Ziegler E, Johnson H, *et al.* (2017, May 21): Nipype: a flexible, lightweight and extensible neuroimaging data processing framework in Python. 0.13.1, version 0.13.1. Zenodo. https://doi.org/10.5281/zenodo.581704

4. Tustison NJ, Avants BB, Cook PA, Zheng Y, Egan A, Yushkevich PA, Gee JC (2010): N4ITK: improved N3 bias correction. *IEEE Trans Med Imaging* 29: 1310–1320.

5. Avants BB, Epstein CL, Grossman M, Gee JC (2008): Symmetric diffeomorphic image registration with cross-correlation: evaluating automated labeling of elderly and neurodegenerative brain. *Med Image Anal* 12: 26–41.

6. Zhang Y, Brady M, Smith S (2001): Segmentation of brain MR images through a hidden Markov random field model and the expectation-maximization algorithm. *IEEE Trans Med Imaging* 20: 45–57.

7. Dale AM, Fischl B, Sereno MI (1999): Cortical Surface-Based Analysis: I. Segmentation and Surface Reconstruction. *NeuroImage* 9: 179–194.

8. Klein A, Ghosh SS, Bao FS, Giard J, Häme Y, Stavsky E, *et al.* (2017): Mindboggling morphometry of human brains. *PLOS Computational Biology* 13: e1005350.

9. Glasser MF, Sotiropoulos SN, Wilson JA, Coalson TS, Fischl B, Andersson JL, *et al.* (2013): The minimal preprocessing pipelines for the Human Connectome Project. *Neuroimage* 80: 105–124.

10. Ciric R, Thompson WH, Lorenz R, Goncalves M, MacNicol EE, Markiewicz CJ, *et al.* (2022): TemplateFlow: FAIR-sharing of multi-scale, multi-species brain models. *Nat Methods* 19: 1568–1571.

11. Evans AC, Janke AL, Collins DL, Baillet S (2012): Brain templates and atlases. *Neuroimage* 62: 911–922.

12. Fonov V, Evans A, McKinstry R, Almli C, Collins D (2009): Unbiased nonlinear average age-appropriate brain templates from birth to adulthood. *NeuroImage* 47: S102.

13. Jenkinson M, Bannister P, Brady M, Smith S (2002): Improved optimization for the robust and accurate linear registration and motion correction of brain images. *Neuroimage* 17: 825–841.

14. Greve DN, Fischl B (2009): Accurate and robust brain image alignment using boundary-based registration. *Neuroimage* 48: 63–72.

15. Lanczos C (1964): A Precision Approximation of the Gamma Function. *Journal of the Society for Industrial and Applied Mathematics Series B Numerical Analysis* 1: 86–96.

16. Abraham A, Pedregosa F, Eickenberg M, Gervais P, Mueller A, Kossaifi J, *et al.* (2014): Machine learning for neuroimaging with scikit-learn. *Front Neuroinform* 8. https://doi.org/10.3389/fninf.2014.00014

17. Power JD, Plitt M, Kundu P, Bandettini PA, Martin A (2017): Temporal interpolation alters motion in fMRI scans: Magnitudes and consequences for artifact detection ((X.-N. Zuo, editor)). *PLoS ONE* 12: e0182939.

18. Brett M, Leff AP, Rorden C, Ashburner J (2001): Spatial Normalization of Brain Images with Focal Lesions Using Cost Function Masking. *NeuroImage* 14: 486–500.

19. Whitfield-Gabrieli S, Nieto-Castanon A, Ghosh S (2011): Artifact detection tools (ART). *Cambridge, MA Release Version* 7: 11.

20. Nieto-Castanon A (2022, October 24): Preparing fMRI Data for Statistical Analysis [no. arXiv:2210.13564]. arXiv. https://doi.org/10.48550/arXiv.2210.13564

21. Power JD, Mitra A, Laumann TO, Snyder AZ, Schlaggar BL, Petersen SE (2014): Methods to detect, characterize, and remove motion artifact in resting state fMRI. *NeuroImage* 84: 320–341.

22. Nieto-Castanon A (2020): *Handbook of Functional Connectivity Magnetic Resonance Imaging Methods in CONN*. Hilbert Press.

23. Friston KJ, Williams S, Howard R, Frackowiak RS, Turner R (1996): Movement-related effects in fMRI time-series. *Magn Reson Med* 35: 346–355.

24. Hallquist MN, Hwang K, Luna B (2013): The nuisance of nuisance regression: Spectral misspecification in a common approach to resting-state fMRI preprocessing reintroduces noise and obscures functional connectivity. *NeuroImage* 82: 208–225.

25. Behzadi Y, Restom K, Liau J, Liu TT (2007): A component based noise correction method (CompCor) for BOLD and perfusion based fMRI. *NeuroImage* 37: 90–101.

26. Chai XJ, Castañón AN, Öngür D, Whitfield-Gabrieli S (2012): Anticorrelations in resting state networks without global signal regression. *NeuroImage* 59: 1420–1428.

27. Deed - CC0 1.0 Universal - Creative Commons (n.d.): Retrieved July 30, 2025, from https://creativecommons.org/publicdomain/zero/1.0/
